# *In Situ* Cultivation Allows for Recovery of Bacterial Types Competitive in Their Natural Environment

**DOI:** 10.1264/jsme2.ME16079

**Published:** 2016-09-29

**Authors:** Dawoon Jung, Yoshiteru Aoi, Slava S. Epstein

**Affiliations:** 1 Institute for Sustainable Sciences and development, Hiroshima University2–313 Kagamiyama, Higashi-Hiroshima, Hiroshima, 739–8511Japan; 2 Department of Biology, Northeastern University360 Huntington Ave, 305 Mugar Life Sciences, Boston, MA 02115USA

**Keywords:** diffusion chamber, cold adaptation, arctic microbes, growth characteristics

## Abstract

Standard cultivation fails to grow most microorganisms, whereas *in situ* cultivation allows for the isolation of comparatively diverse and novel microorganisms. Information on similarities and differences in the physiological properties of isolates obtained from *in situ* cultivation and standard cultivation is limited. Therefore, we used the arctic sediment samples and compared two culture collections obtained using standard and novel cultivation techniques. Even though there was no temperature selection at the isolation step, isolates from each method showed different reactions to temperature. The results of the present study suggest that isolates from *in situ* cultivation are more competitive in their natural environment.

Conventional cultivation leads to the isolation of only a small fraction of environmental species, which are often rare and of unclear relevance to the community function (1, 6, 8). A group of methods based on *in situ* cultivation is becoming prominent with the recent surge of new cultivation strategies (2, 3, 5, 7, 9–12).

These methods are all based on one underlying concept: nature contains all growth factors necessarily for microbial growth. This simple observation may be utilized to grow species with unknown growth requirements by enclosing them in an entrapment with semi-permeable walls. If these entrapments are incubated *in situ*, permeability may allow diffusion to equilibrate the chemical milieu inside and outside, such that cells “trapped” inside have access to their naturally occurring growth components. Historically first was a simple diffusion chamber (10), which led to the recovery of 300-fold more colonies with greater phylogenetic novelty than a standard Petri dish. However, it currently remains unclear whether these isolates have superior environmental fitness to the “standard” isolates. Thus, the only strains that grow in diffusion chambers are those capable of growth *in situ*, whereas those that grow in Petri dishes may originate from inactive spores, dormant cells, or transient members of the community. If this is the case, diffusion chamber-reared isolates may be more adapted to the conditions of their original environment than those grown using standard cultivation from the same habitat. We herein examined this hypothesis by comparing the temperature preferences of isolates obtained by the diffusion chamber approach *vs.* those grown with conventional techniques using Northern Greenland as the test environment. The null hypothesis is that the former will be better adapted to cold temperatures than the latter.

In July 2010, we collected lake sediment in the vicinity of Illulisat, Greenland, at 69.2°N, 51.0°W, and transported it on ice to the home laboratory at Northeastern University, Boston MA, USA. The extant temperature at the time of sampling was 10°C; summer temperatures in this environment typically fluctuate between 5–20°C. In the lab, 1-g samples of sediment were diluted 10^−4^ to 10^−6^ with Nutrient broth (Difco), at 1% of the concentration suggested by the manufacturer, and 1.5% Bacto agar (Difco). These serial dilutions were inoculated into diffusion chambers as described previously (4, 5), which were then returned to the sediment samples for incubation. In parallel to diffusion chamber cultivation, the same serial dilutions were plated on standard Petri dishes with agar supplemented as above, then incubated at 10°C. The chambers were removed after a 4-weeks incubation at 10°C, their contents were homogenized using a sterile stick, vortexed, diluted with sterile water, and then plated on the above agar medium. The taxonomic positions of 30 strains were identified on the basis of 16S rRNA gene sequences. In the same manner, we identified 30 microbial isolates from standard Petri dishes. The 16S rRNA gene was amplified using the universal primers 27F and 1492R, and partial sequences of 690–789 bp were compared with those available in the GenBank (www.ncbi.nlm.nih.gov) database using the MEGA program (13) in order to identify their closest relatives. Isolates were classified into 11 species defined as clusters of sequences sharing >97% homology in their 16S rRNA gene sequences. Some of these clusters were represented by a single cultivated strain, while others contained multiple isolates, and we selected 17 of these isolates to represent 11 species for further investigations (Table 1); several were found to be isolated exclusively by the diffusion chamber approach (DC group), while the others were mostly, but not exclusively isolated by standard cultivation (SA group; several strains isolated by the diffusion chamber approach were classified as SA species). Therefore, in some cases, this choice led to the testing of strains within a given species that were obtained by different methods (*Pseudomonas mandeli*), whereas in others (*Pseudomonas lini*), the strains were obtained by the same (DC) method. The experimental design is shown in Fig. 1.

Isolate temperature preferences and tolerances were evaluated in three ways. The abilities of the isolates to grow on solid medium (same as above) were examined in triplicate at 10, 22, and 30°C. All isolates formed colonies at 10°C within four d. At 30°C, 100% of SA isolates grew, whereas 78% of DC isolates did not (Table 2). We then tested the high temperature tolerances of the isolates by exposing them to 37°C for 72 h and then assessing their viabilities on agar plates (as above) at 22°C. All, except for two SA isolates recovered, while none of the DC isolates survived the treatment (Table 2). The two SA isolates that did not tolerate the heat exposure are on the list of organisms co-isolated by both isolation methods (diffusion chamber and standard cultivation). We subsequently measured the growth rates of all isolates in triplicate by following the optical density of liquid cultures at 0, 4, 10, 16, 22, 28, 34, and 37°C. One hundred-fold diluted nutrient broth was inoculated with 5–20 μL of a cell suspension from overnight liquid cultures grown at 10°C and was then incubated with shaking at 200 rpm. The specific growth rate *μ* was calculated after establishing growth curves using the non-linear regression function of Sigma plot software (Systat Software).

The results obtained are presented in Fig. 2. None of the isolates appeared to be psychrophilic, and most achieved the highest specific growth rate at 22°C. This temperature only occasionally occurred in the sampled area, and most species present in summer may quickly adapt to take advantage of these episodes. While the isolates tested all appeared to be mesophiles, marked differences were observed between their adaptation to the extremes of the temperature range they were subjected to. All SA isolates grew at 34°C, whereas none of the DC group did (Fig. 3). The growth rate of SA isolates gradually decreased to its 0°C minimum, whereas that of DC isolates remained invariable between 10 and 0°C. This result suggests that DC strains are not temperature-limited in cold environments, which is in contrast to SA isolates, suggesting that they have specific cold-related adaptations that are lacking in the SA culture collection.

In summary, isolates obtained by the diffusion chamber approach differed from those grown by standard techniques in every aspect investigated. The differences identified indicate that the first either have particular adaptations for growth at low temperatures, lack adaptations to tolerate high temperatures, or both. This result supports the original hypothesis that the diffusion chamber approach and, by extension, other *in situ*-based cultivation methods result in the isolation of species that are more competitive in their community than those obtained by conventional cultivation.

The partial sequences of the 16S rRNA gene obtained for 17 isolates of interest were deposited in GenBank with the accession numbers KX094424 to KX094440.

## Figures and Tables

**Fig. 1:**
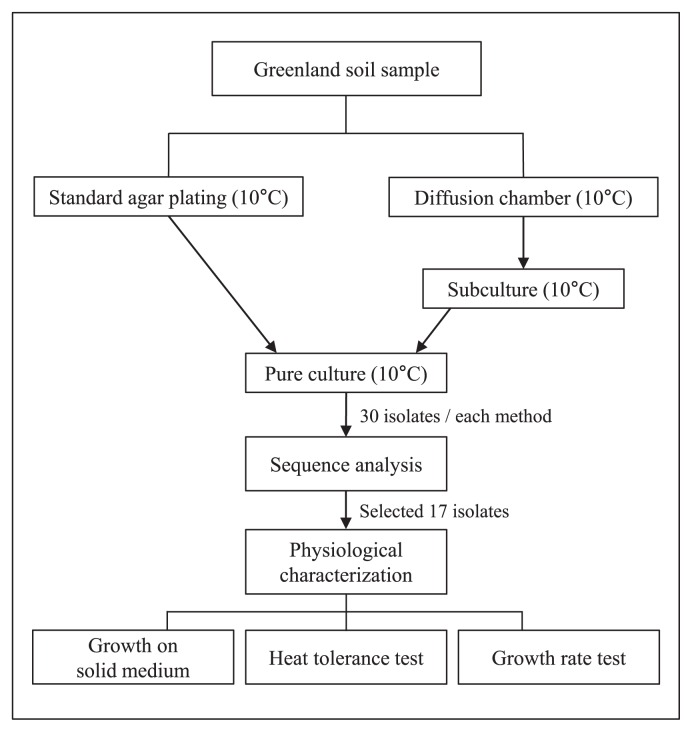
Flowchart of the experimental design.

**Fig. 2:**
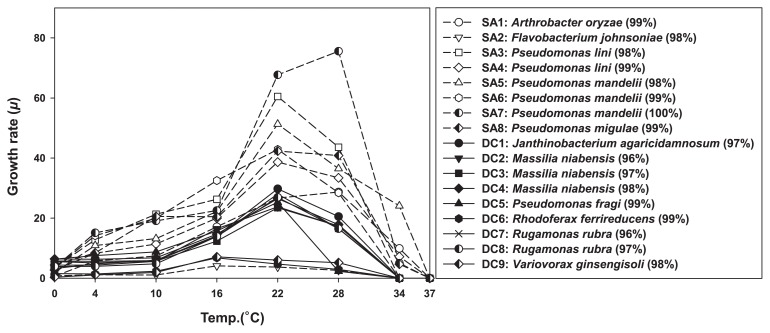
Growth rates of DC (diffusion chamber; solid line) and SA (standard agar plating; dash line) isolates at different temperatures. The right panel shows a list of the tested isolates with the names of their closest relatives from among validly described species, as obtained by 16S rRNA gene identities.

**Fig. 3:**
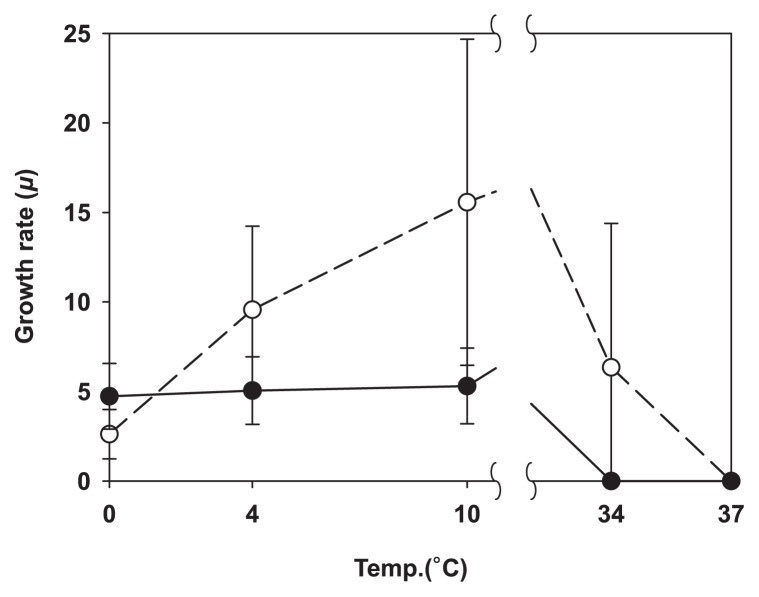
Specific growth rate *μ* at selected temperatures; the solid circle is the average for DC, and the blank circle is the average for SA isolates. The error bars represent the standard deviation of the mean value.

**Table 1:** Phylogenetic affiliations of isolates with standard agar plating and diffusion chamber methods on the basis of 16S rRNA gene sequences

Method	Taxonomic group	Closest species	Similarity (%)	No. of isolates
Standard agar plating	*Actinobacteria*	*Arthrobacter oryzae*	98–99	4
	*Flavobacteria*	*Flavobacterium johnsoniae*	98	5
	*Gammaproteobacteria*	*Pseudomonas migulae*	99	2
		*Pseudomonas lini*	98–99	13
		*Pseudomonas mandelii*	100	6

Diffusion chamber	*Betaproteobacteria*	*Janthinobacterium agaricidamnosum*	97	1
		*Massilia niabensis*	96–98	8
		*Rhodoferax ferrireducens*	99	1
		*Variovorax ginsengisoli*	98	1
	*Gammaproteobacteria*	*Pseudomonas fragi*	99	8
		*Pseudomonas lini*	99	1
		*Pseudomonas mandelii*	98–100	7
		*Rugamonas rubra*	96–97	3

**Table 2:** Growth of isolates on agar plates (10, 22, and 30°C) and recovery from heat shock (at 37°C for 72 h)

Group	Isolate	Closest species	Method	Growth on agar plate			Heat tolerance at 37°C

				10°C	22°C	30°C	
Standard agar plating	SA1	*Arthrobacter oryzae*^a^	SA	+	+	+	+
	SA2	*Flavobacterium johnsoniae*^a^	SA	+	+	+	+
	SA3	*Pseudomonas lini*^b^	SA	+	+	+	−
	SA4	*Pseudomonas lini*	SA	+	+	+	+
	SA5	*Pseudomonas mandelii*^b^	DC	+	+	+	+
	SA6	*Pseudomonas mandelii*	DC	+	+	+	−
	SA7	*Pseudomonas mandelii*	SA	+	+	+	+
	SA8	*Pseudomonas migulae*^a^	SA	+	+	+	+

Diffusion chamber	DC1	*Janthinobacterium agaricidamnosum*^c^	DC	+	+	−	−
	DC2	*Massilia niabensis*^c^	DC	+	+	−	−
	DC3	*Massilia niabensis*	DC	+	+	−	−
	DC4	*Massilia niabensis*	DC	+	+	−	−
	DC5	*Pseudomonas fragi*^c^	DC	+	+	+	−
	DC6	*Rhodoferax ferrireducens*^c^	DC	+	+	−	−
	DC7	*Rugamonas rubra*^c^	DC	+	+	−	−
	DC8	*Rugamonas rubra*	DC	+	+	−	−
	DC9	*Variovorax ginsengisoli*^c^	DC	+	+	+	−

a Species only observed on standard agar plates

b Species detected by the SA and DC methods

c Species only observed among diffusion chamber isolates
